# Identifying the Relative Priorities of Subpopulations for Containing Infectious Disease Spread

**DOI:** 10.1371/journal.pone.0065271

**Published:** 2013-06-12

**Authors:** Shang Xia, Jiming Liu, William Cheung

**Affiliations:** Department of Computer Science, Hong Kong Baptist University, Hong Kong S.A.R; University of Zaragoza, Spain

## Abstract

In response to the outbreak of an emerging infectious disease, e.g., H1N1 influenza, public health authorities will take timely and effective intervention measures to contain disease spread. However, due to the scarcity of required resources and the consequent social-economic impacts, interventions may be suggested to cover only certain subpopulations, e.g., immunizing vulnerable children and the elderly as well as closing schools or workplaces for social distancing. Here we are interested in addressing the question of how to identify the relative priorities of subpopulations for two measures of disease intervention, namely vaccination and contact reduction, especially when these measures are implemented together at the same time. We consider the measure of vaccination that immunizes susceptible individuals in different age subpopulations and the measure of contact reduction that cuts down individuals’ effective contacts in different social settings, e.g., schools, households, workplaces, and general communities. In addition, we construct individuals’ cross-age *contact frequency matrix* by inferring *basic contact patterns* respectively for different social settings from the socio-demographical census data. By doing so, we present a prioritization approach to identifying the target subpopulations that will lead to the greatest reduction in the number of disease transmissions. We calculate the relative priorities of subpopulations by considering the marginal effects of reducing the reproduction number for the cases of vaccine allocation by age and contact reduction by social setting. We examine the proposed approach by revisiting the real-world scenario of the 2009 Hong Kong H1N1 influenza epidemic and determine the relative priorities of subpopulations for age-specific vaccination and setting-specific contact reduction. We simulate the influenza-like disease spread under different settings of intervention. The results have shown that the proposed approach can improve the effectiveness of disease control by containing disease transmissions in a host population.

## Introduction

In controlling the spread of an emerging infectious disease, public health authorities need to take timely and effective disease intervention measures [1], [2]. Some examples of the measures are vaccine allocation that immunizes susceptible individuals [3], social distancing that reduces individuals effective contacts accounting for disease transmissions [4], and the prophylactic use of antiviral drugs and the prompt treatment of infections that change the susceptibility and infectivity of uninfected and infected individuals, respectively [5]. In practice, it would be difficult to excise such measures to cover a majority of the host population due to the scarcity of required resources, e.g., the potential shortage of vaccine supply and the inadequate stockpiles of antiviral drugs [6], [7], as well as the consequent social-economic impacts, e.g., school closure and workplace shutdown [8], [9]. As a result, public health authorities will select certain target subpopulations for disease interventions. For instance, during the 2009 Hong Kong H1N1 influenza epidemic, the government announced the immediate closure of all primary schools, kindergartens when the first non-imported case was confirmed and meanwhile targeted children between the ages of 6 months and below 6 years as the priority groups for vaccination [10], [11]. Correctly identifying the target subpopulations for applying intervention measures can be critical for achieving the intended effects of disease control. In this study, we are interested in addressing the question of how to decide the relative priorities of subpopulations for some measures of disease intervention during the time course of disease spread. Specifically, the measures to be considered are vaccine allocation, contact reduction, and the combination of both.

Vaccination has been regarded as one of the most effective methods for disease control due to the *herd immunity effect* (i.e., by immunizing a certain portion of the host population, it will provide indirect protections for the unimmunized individuals [12], [13]). Existing studies that focused on vaccine allocation in an age-structured population have suggested different criteria for targeting priority subpopulations, e.g., school-age children for preventing disease transmissions [3], [4], [15], and the elderly for reducing influenza-attributable morbidity and mortality [16], [17]. Moreover, Medlock et al. [18] have found that optimal vaccine allocation (i.e., by minimizing hospitalizations, total attack rate, and deaths) among people in different age groups should be performed in relation to the state of epidemic at the time when vaccine doses become available. Matrajt et al. [19] have showed that target populations for vaccination may switch from transmissible individuals to infection-vulnerable ones at the time near the infection peak. Furthermore, Wallinga et al. in [20] have related the identification of vaccination target groups with the present situation of disease prevalence, e.g., the group-specific force of infection and incidence rates. All these mentioned studies have considered the priority subpopulations for vaccine allocation according to their adopted measurements of vaccination effectiveness. In the real world, vaccination is unlikely to be adopted alone. That is to say, the identification of priority subpopulations will be affected by the implementation of other intervention measures at the same time, e.g., cutting down individuals effective contacts for reducing disease transmissions.

Disease transmissions are subject to the structure of individuals social contacts, which plays a key role in the assessment of an infection outbreak [21]. For this reason, previous efforts have been made to characterize the social contact relationships based on the empirical data of individuals contact frequencies and durations [22], [23]. For instance, Mossong et al. [24] have surveyed individuals daily contact activities and observed the highly age-related *patterns* of individuals actual contacts. By representing the contacts using a *contact frequency matrix*, they have shown that there exist strong diagonal elements among those aged 5–24 years, which indicate that individuals tend to mix with others of similar ages within places such as schools. At the same time, there also appear parallel secondary diagonals that represent children mixing with adults mainly through households, and a wider contact “plateau among adults that accounts for contacts occurring in workplaces. Individuals cross-age contacts exhibit specific patterns that correspond to the likelihoods of individuals mixing together within certain social settings (i.e., schools, households, workplaces, or general communities), which in turn depend on the socio-demographical structure of the population (i.e., age distribution, school attendance, household size, and working population, etc.). This suggests that disease transmissions through social contacts are mainly attributed to the transmissions occurring in some typical social contact settings. In addition, individuals social contacts may change due to either individuals self-initiated behaviors (e.g., avoidance of public places) or governmental compulsory policies (e.g., school closures and workplace shutdown). In this work, we evaluate the change of social contacts based on the change of individuals contacts occurring in different social settings. Thus, disease intervention by contact reduction in the host population refers to the adjustment of the ratios of individuals contacts in such social settings.

The aim of this study is to propose an approach to identifying the relative priorities of subpopulations for disease interventions by age-specific vaccine allocation and setting-specific contact reduction. This approach utilizes some prior knowledge about individuals age-specific susceptibility and infectivity, disease prevalence at the time of intervention implementation, and the basic patterns of individuals contacts within each social setting. While, it does not rely on detailed information about individuals actual social contacts, nor their potential changes in response to disease spread, which could be difficult if not impossible to obtain timely and precisely. We infer individuals setting-specific contact patterns from the socio-demographical census data of the considered population. The overall social contacts that account for disease transmissions will be estimated by incorporating the basic setting-specific contact patterns with the coefficients corresponding to the ratios of individuals contacts within different social settings. We evaluate the effects of disease interventions for containing disease transmissions by measuring the reproduction number with respect to the susceptible population sizes and individuals contact frequencies. By doing so, we prioritize the subpopulations that will lead to the greatest reduction in the number of disease transmissions by means of considering the marginal effects of reducing the reproduction number for the cases of vaccine allocation by age and contact reduction by social setting.

In the study, for the sake of demonstration, we develop a compartmental model to describe the dynamics of the influenza-like disease spread among an age-structured host population. We generate individuals setting-specific contact matrices from the socio-demographical census data of Hong Kong. We parameterize the disease model with the epidemiological data from the 2009 Hong Kong H1N1 influenza epidemic. Based on such a parameterization, we implement the proposed approach to identifying the relative priorities of subpopulations for disease interventions in Hong Kong. Additionally, we carry out a series of simulations with different settings of disease intervention. The results have shown that the proposed prioritization of age subpopulations and/or social settings can improve the effectiveness of disease control by containing disease transmissions in the host population.

## Methods

In this section, we first develop a compartmental model that describes the dynamics of an influenza-like disease spread in an age-structured population. We then characterize disease transmissions by using the reproduction matrix. Finally, we examine the prioritization of subpopulations by evaluating the effects of disease intervention on the containment of disease transmissions. The parameters that will be used in this section are listed in Table 1.

**Table 1:** The list of parameters used for modeling.

Symbol	Meaning (a subscript denotes a certain subpopulation by age)
*N*	number of age-specific subpopulations
*S_i_* (t)	susceptible population size
*I_i_* (t)	infectious population size
*R_i_* (t)	recovered population size
*P_i_*	overall population size
*α*_i_	infectivity
*β_i_*	susceptibility
*λ_i_*	infection rate
*μ*	disease transmission rate
*γ*	recovery rate
*c_ij_*	cross-age contact frequency
*R_0_*	basic reproduction number
*R_t_*	effective reproduction number
*C_H_*	household contact pattern
*C_S_*	school contact pattern
*C_W_*	workplace contact pattern
*C_C_*	community contact pattern
*C*	actual contact matrix
*r_H_*	household contact pattern coefficient
*r_S_*	school contact pattern coefficient
*r_W_*	workplace contact pattern coefficient
*r_C_*	community contact pattern coefficient
*K*	reproduction matrix
*A*	infectivity matrix, *diag* (*α*_1_,…,*α_N_*)
*B*	susceptibility matrix, *diag* (*β*_1_,…,*β_N_*)
*S*	susceptible population matrix, *diag* (*S*_1_ (t),…,*S_N_* (t))
*I*	infectious population vector, {*I*_1_ (t)…*I_N_* (t)}*^T^*
*w*_1_	top right eigenvector of *K*
*v*_1_	top left eigenvector of *K*

### Disease Model

We now introduce a standard susceptible-infectious-recovered (SIR) model to characterize the dynamics of infectious disease spread, in which individuals are divided into 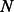 subpopulations with reference to their ages. Each individual in age group 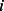 belongs to one of three infection associated compartments: susceptible (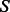), infectious (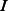), and recovered/immunized (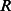). Correspondingly, the number of individuals in each compartment at time step 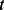 is denoted by 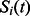, 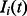, and 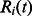, respectively. We consider the spread of an infectious disease within a single circulation season, such that the natural birth and death of the population are not taken into account. The total number of individuals in age group 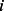, denoted by 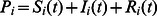, is static.

The dynamics in the time course of disease spread is described by the following set of differential equations:
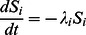


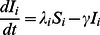
(1)

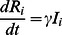
Here, 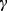 represents the rate of recovery corresponding to the duration of disease infection. 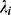 is the infection rate that denotes the probability of being infected for susceptible individuals in age group 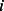. For time unit 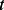, 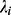 can be calculated as follows:
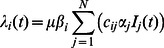
(2)where 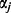 measures the infectivity for individuals in group 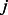, which is the probability of transmitting disease when an infectious individual contacts with other susceptible individuals. 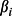 denotes the susceptibility for individuals in group 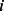, which represents the probability of being infected when a susceptible individual is exposed to infectious contacts. 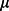 is a constant disease transmission rate for all age groups and can be estimated from 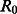 in the initial stage of disease spread.

We consider the susceptible individuals being infected through their contacts with the infectious ones. The number of disease transmissions among different age groups is therefore determined by the frequencies of contacts among them. Here, we use 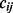 to describe the contact frequency between a pair of individuals in age groups 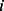 and 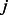. 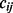 is calculated as the total number of contacts between two age groups, 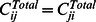, divided by the product of their population sizes, 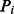 and 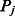:
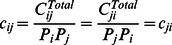
(3)Matrix 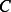 with the elements of 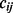 is the overall contact matrix that describes individuals cross-age contact frequencies. Based on the definition, matrix 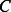 is symmetric for 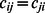.

In view of the lack of accurate data for characterizing individuals actual contacts, we adopt a computational approach to inferring the setting-specific *contact patterns* from the available socio-demographical census data [25], [26]. In doing so, we calculate the probability for individuals of different ages mixing together within certain social settings, i.e., individuals sharing the same places, such as households, schools, workplaces, and general communities. Then, we generate four matrices accounting for the specific patterns of individuals contacts within each social setting, which is represented by 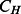 for contacts within households, 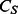 for schools, 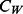 for workplaces, and 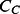 for general communities, respectively. Thus, we can estimate the overall matrix of individuals cross-age contact frequencies as a linear combination of the four setting-specific matrices:
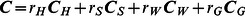
(4)where coefficients 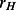, 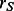, 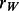 a
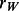
d 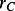 denote the ratios of individuals contacts occurring in the above-mentioned social settings, respectively, and their sum is equal to one:
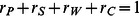
(5)


### Reproduction Matrix

With respect to generations of disease infection, we can use the reproduction matrix 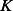 (i.e., also known as *next generation matrix* (NGM) [27]–[29]) to rewrite the equations of disease transmission dynamics as follows:
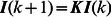
(6)where vector 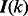 with elements 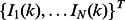 denotes the number of infectious individuals in each age group at the 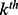 generation of disease infection. For the aforementioned 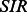 model, the reproduction matrix 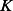 can be calculated as follows:



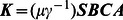
(7)Here, matrix 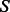 describes the sizes of susceptible populations in each age group; it has elements 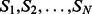 in the diagonal and zeros elsewhere. Matrix 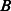 summarizes individuals age-specific susceptibility with the diagonal elements of 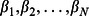 and zeros elsewhere. Matrix 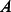 gives the age-specific infectivity of infected individuals with the diagonal elements of 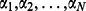 and zero elsewhere. Moreover, during the course of disease transmission, the susceptible population sizes will decrease over time, and therefore matrices 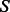 and 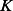 are dependent on time 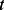. In what follows, the variables of 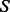 and 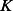 have meanings similar to 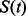 and 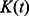.

In epidemiology, reproduction number 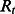 refers to the number of newly infection cases caused by a typical infectious individual in a completely susceptible population [27], [30]. By constructing the next-generation-matrix [27]–[29], reproduction number 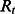 in the context of an age-structured host population can be approximately estimated as the dominant eigenvalue of reproduction matrix 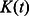:
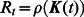
(8)


Given the condition that matrices 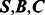 and ***A*** are all symmetric, reproduction number 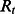 can be approximately calculated as:
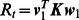
(9)where 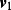 and 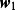 are the top left and right eigenvectors of reproduction matrix 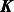 (i.e., the corresponding top eigenvalue is 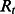). Specifically, we choose a normalized format of each eigenvector in which the elements are positive and sum up to one. As proposed by Wallinga et al. in [20], 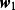 and 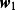 can be approximately correlated to the number of new infections in each age group at generation 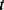, 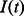:



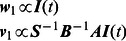
(10)Therefore, we can describe disease transmissions (i.e., reproduction number 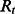) with reference to the present disease prevalence, the susceptible population sizes, and their cross-age contact frequencies. In what follows, we will examine the effects of disease interventions (i.e., vaccination and contact reduction) on containing disease transmissions.

### Effects of Intervention Measures

In this section, we evaluate the effects of disease interventions (i.e., age-specific vaccination, setting-specific contact reduction, or both) for containing disease transmissions by measuring the marginal reduction of reproduction number 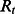.

#### Vaccination

As aforementioned, disease transmissions can be estimated based on 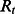, which is correlated to reproduction matrix 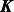. Thus, the change of the reproduction number, 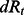, can be calculated from 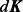 as follows:
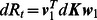
(11)


Vaccination that immunizes susceptible individuals can reduce the susceptible population sizes in different age groups. The reduction of 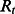 due to vaccination will be proportional to the following terms:
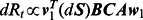
(12)


Specifically, when targeting a susceptible population in age group *i*, the effects of vaccination can be calculated as follows:
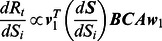
(13)


By combining the elements of each matrix, we arrive at an indicator that evaluates the marginal reduction of 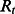 by vaccinating a unit of susceptible individuals in age group *i*:
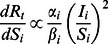
(14)


Due to the lack of knowledge about the report rate of disease infections, i.e., the ratio of confirmed cases to the overall infections, we can approximately estimate the susceptible population size 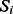 using the population size 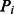, based on the assumption that the number of infections is relatively small in the host population. Therefore, we can prioritize each age group as follows:
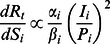
(15)


With the above equation, we can determine the relative priorities of age groups for vaccine allocation with respect to their age-specific infectivity, susceptibility, population sizes, and present disease prevalence.

#### Contact reduction

As for containing disease transmissions by reducing the effective contacts in a host population, we will examine the change of the reproduction number, 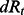, corresponding to the reduction of individuals contact frequencies, 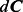:
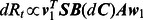
(16)


We examine the effects of contact reduction in terms of cutting down the ratio of individuals contacts within a social setting, 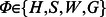:
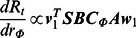
(17)


Based on the inferred setting-specific contact matrices, the relative priority for contact reduction that targets social setting 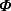 can be computed as follows:
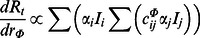
(18)


With the above equation, we can estimate the relative priorities of social settings for contact reduction with respect to individuals age-specific infectivity, susceptibility, present disease prevalence, and their setting-specific contact patterns.

#### Two intervention measures

Next, we consider the case of a campaign against the spread of an infectious disease, in which multiple intervention measures will be implemented at the same time. In view of this, we are interested in exploring the impacts of vaccination and contact reduction being implemented simultaneously. We estimate the marginal reduction of the reproduction number, 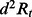, corresponding to both the vaccination of susceptible population 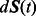 and the reduction of effective social contacts 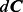:
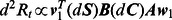
(19)


Specifically, when selecting age group 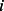 for vaccination and social setting 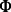 for contact reduction, the impacts of implementing these two intervention measures for reducing reproduction number 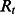 can be evaluated as follows:
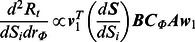
(20)That is,



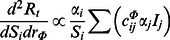
(21)Therefore, by evaluating the interplays of two intervention measures, we can identify the relative priorities of age groups and social settings for vaccine allocation and contact reduction being implemented simultaneously. That is to say, the number of vaccine doses allocated to each age group will be proportional to the relative priority of that group. The contact reduction targets the social setting with the highest priority.

## Results

### The 2009 Hong Kong H1N1 Influenza Epidemic

In order to examine our proposed approach, we revisit the real-world scenario of the 2009 Hong Kong H1N1 influenza epidemic [11], [31]–[33]. Based on the socio-demographical census data of Hong Kong, we divide the host population between 0 and 85+ years of age into 18 groups [26]. The disease parameters used for the modeling and simulation are listed in Table 2. Specifically, the reproduction number is estimated to be 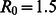 in the initial stage of disease spread [32]. The infectivity is 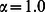, which is homogeneous for all age groups. The susceptibility is estimated to be 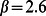 for individuals below 20 years old, who are more susceptible than the rest of the population [11]. The duration of H1N1 influenza infection is set to be 3.2 days [32], [34]. Therefore, the recovery rate is calibrated as 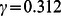 (i.e., 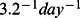) based on the assumption of the exponential distribution of individuals recovery from disease infection.

**Table 2:** The disease parameters as used in the modeling and simulation are calibrated according to the epidemiological data of the 2009 Hong Kong H1N1 influenza epidemic [11], [31], [32], [34].

Parameters		Values	Sources
*R*_0_	reproduction number	1.5	[32]
*α*_ i_	infectivity	1.0	
*β*_i_	susceptibility	2.6 for 0 – 19 *y*	[11]
		1.0 for others	
γ	recovery rate	0.3125 (3.2^−1^ *day* ^−1^)	[32], [34]

**Figure 1:**
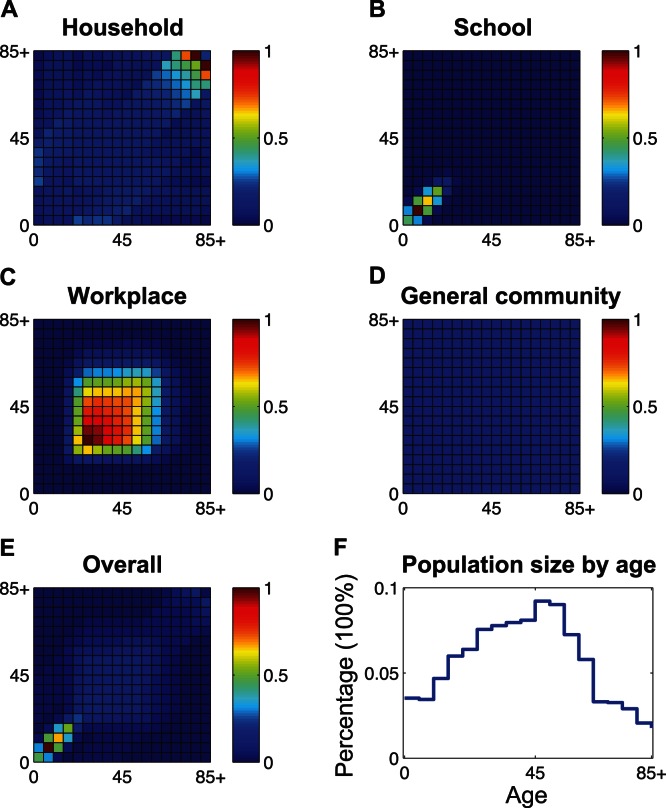
Contact patterns inferred from the socio-demographical census data of Hong Kong. We consider disease transmissions among individuals between 0 and 85+ years old and divide them into 18 age groups. The contact matrices are generated corresponding to the likelihoods of individuals mixing together within respective social settings: **A** household (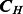), **B** school (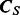), **C** workplace (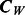), **D** general community (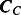). **E** The overall contact matrix is calculated as the linear combination of the four setting-specific contact matrices. The combination coefficient of each matrix denotes the ratio of effective contacts occurring in that social setting. **F** The population size in each age group.

We infer individuals setting-specific contact patterns from the available socio- demographical census data of Hong Kong by calculating the likelihoods of individuals mixing together within different social setting [25], [26]. The generated setting-specific contact pattern matrices are shown in Figure 1. Specifically, Figure 1.a describes the contacts in households, in which the main diagonal and two secondary diagonals correspond to the contacts among couples as well as between parents and children. Figure 1.b shows the pattern of contacts in schools, in which the strong diagonal elements among individuals below 20 years old indicate that students are more inclined to mix with the same age individuals. Figure 1.c presents the pattern of contacts in workplaces, in which the contacts are more frequent among individuals aged between 20 and 65 years old. Figure 1.d gives the pattern of individuals random contacts with each other in general communities. We normalize the elements of the four generated contact pattern matrices so that their total numbers of contacts are equal. As for the overall contact matrix (see Figure 1.e), the coefficients used for combining the setting-specific matrices, 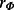 and 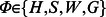, can be approximately estimated to be the fraction of disease infections occurring in the respective social settings. A previous study has shown that 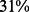 of infections occurred in households during the 2009 Hong Kong H1N1 influenza epidemic [35]. In addition, we assume that the other three contact matrix coefficients follow the empirical estimation of disease infections in the different social settings [2], [21], [25]: 0.24 in schools, 0.16 in workplaces, and 0.29 in general communities.

**Figure 2:**
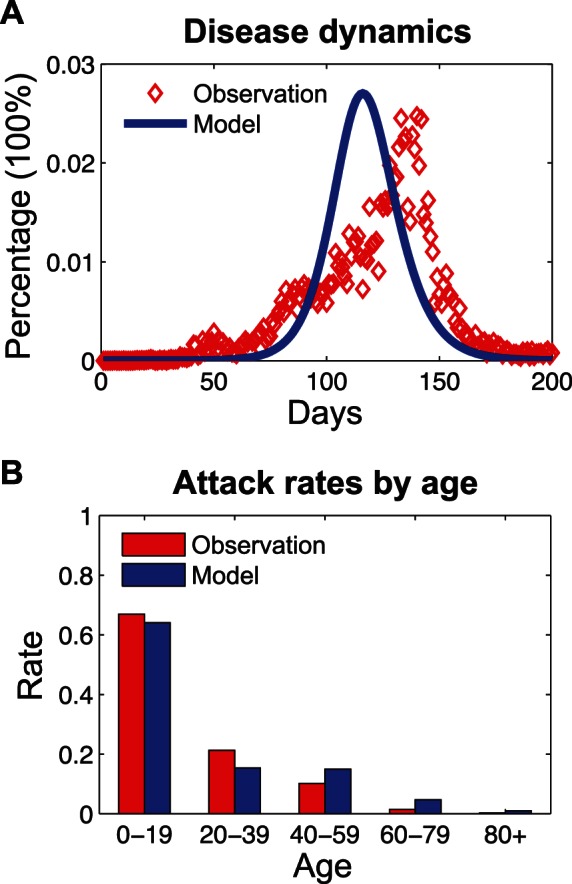
The baseline scenario of disease spread. We calibrate the proposed disease model according to the 2009 Hong Kong H1N1 influenza epidemic. In doing so, we collect the laboratory-confirmed cases of H1N1 infection reported by the Centre for Health Protection (CHP) of Hong Kong Public Health Department for 200 days since the disease onsite in early May 2009 [36]. **A** The temporal dynamics of disease spread in terms of the proportion of the newly infected cases reported each day to the total number of disease infections. **B** A comparison of the observed and estimated age-specific attack rates.

Based on the above parameterization, we validate our disease model by comparing the model predictions with the real-world observations in term of the new infection cases per day and the age-specific attack rates (see Figure 2). In doing so, we collect the laboratory-confirmed cases of H1N1 infection daily reported by the Centre for Health Protection (CHP) of Hong Kong Public Health Department for 200 days since the disease onsite on early May, 2009 [36]. The results of simulation over the specified period show that disease infection peaked around 120 days since the disease onsite (see Figure 2.a) and the young and school-age students (between 0 and 19 years old) constituted a large proportion of the infection cases, while that of adults was relatively small (see Figure 2.b).

### Prioritization of Subpopulations

**Figure 3:**
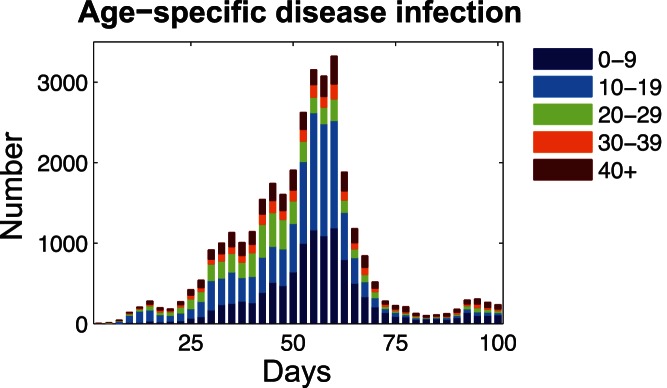
The number of reported infections in different age groups during the spread of H1N1 influenza in Hong Kong. We collected the laboratory-confirmed cases of infection reported by the Centre for Health Protection (CHP) of Hong Kong Public Health Department for 200 days since the disease onsite in early May 2009 [36].

Figure 3 shows the daily number of reported new infections in each age group during the spread of H1N1 influenza in Hong Kong. Furthermore, based on our proposed approach, we examine the relative priorities of subpopulations for disease interventions in Hong Kong.

**Figure 4:**
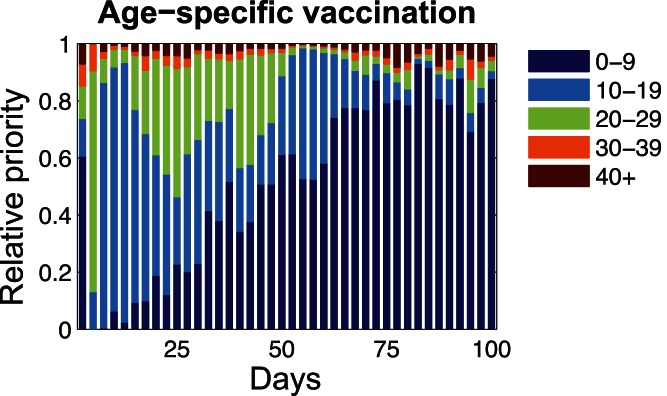
Prioritization of age groups for vaccine allocation during the course of disease spread.

Figure 4 shows the relative priorities of age groups for vaccine allocation during the course of disease spread. Generally speaking, individuals between 0 and 29 years old are the most important subpopulation for containing disease transmissions by means of vaccination. While, for each specific age group, the identified priorities vary in different stages of disease spread. For the first month since the disease onsite, e.g., day 25, we can observe that individuals between 10 and 19 years of age are targeted as the top priority subpopulation for vaccination. This describes the situation in which outbreaks will appear among school-age students due to their high frequency of contacts. Subsequently, on day 50, it can be observed that the relative priorities of individuals aged between 0 and 9 and those between 20 and 29 are increased, while the priorities of individuals between 10 and 19 are relatively decreased. When disease infection peaks near day 120, it is observed that individuals between 0 and 19 will become dominant for vaccination, which agrees with the real-world observation that the children and school-age students accounted for a large proportion of the new infections in this stage (see Figure 3). Finally, in the decay stage of the epidemic, children between 0 and 9 will become the subpopulation with the highest priority for vaccination. However, it should be pointed out that vaccination is more effective in the initial stage of disease spread than in the stage of decay.

**Figure 5:**
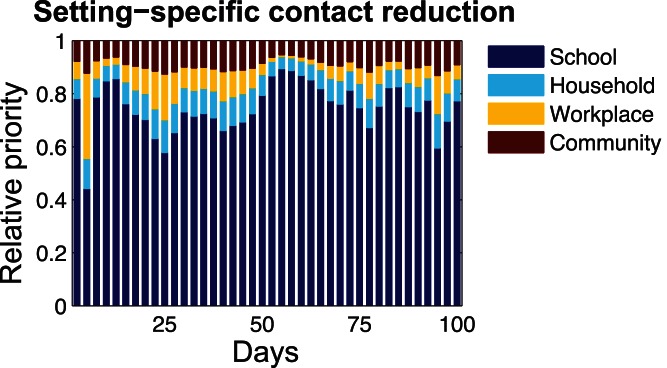
Prioritization of social settings for individuals contact reduction in different stages of disease spread.

Figure 5 shows the relative priorities of social settings for disease intervention by contact reduction among individuals. General speaking, the reduction of individuals contacts within schools is identified as the key measure for containing disease transmissions during the whole period of disease spread. As for households and workplaces, disease transmissions within these two social settings account for a relatively large proportion during the initial and the decay stages of disease spread (i.e., between day 25 and day 100 and between day 150 and day 200, respectively). When infection peaks around day 120, individuals contacts within households and workplaces account for a relatively smaller proportion for disease transmissions. As mentioned before, the estimated proportion of infections occurring in schools was not the largest among the four considered social settings (i.e., the empirical estimation for schools was about 24%, and households 31%). However, the disease infections that had occurred in schools played a significant role for the disease transmissions among the host population. Therefore, the reduction of individuals effective contacts in the social setting of schools (i.e., through measures of school closure as well as methods of school sanitation and disinfection) should be implemented immediately after the disease onsite, lasting for the whole period of disease spread.

**Figure 6:**
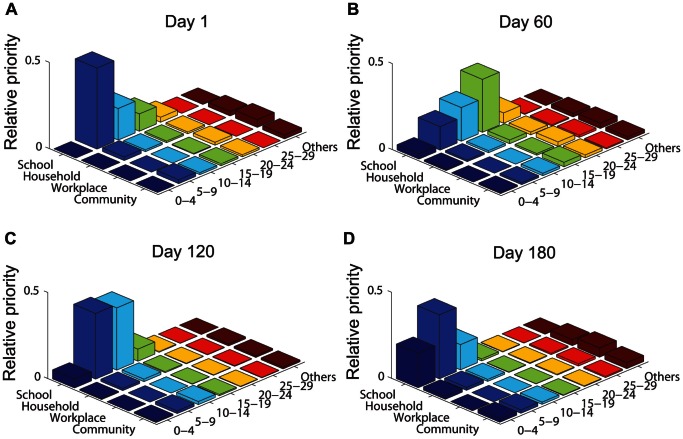
Prioritization of age groups and social settings for implementing age-specific vaccination and setting-specific contact reduction concurrently in different stages of disease spread.

Figure 6 further presents the relative priorities of age groups and social settings for the implementation of vaccine allocation and contact reduction at the same time. Generally speaking, vaccination of individuals between 0 and 19 and contact reduction in schools are the most important measures for containing disease transmissions. As for others of older ages, contact reduction in households, workplaces, and general communities would become relatively more important. Specifically, as shown in Figure 6.a, at the very beginning of disease spread, i.e., on day 1, contact reduction in schools and vaccination of individuals aged between 5 and 19 should be the top priority in order to contain disease transmissions. On day 60, as indicated in Figure 6.b, individuals between 15 and 19 are identified as the target subpopulation for vaccination, followed by individuals aged 10–14 and 5–9. In this stage, contact reduction in the social setting of schools remains to be the top priority. When disease infection peaks around day 120, age groups 5–9 and 10–14 would become the most important subpopulations for vaccination. In the final stage of disease spread, vaccinating children would become more important.

By comparing the results of disease interventions by vaccination only (shown in Figure 4) with those by vaccination and contact reduction simultaneously (shown in Figure 6), we observe that age group 20–29 has a higher priority for vaccine allocation in the case of vaccination only than that in the case of adopting two intervention measures. This is mainly due to the interplay of the two intervention measures in that the effects of reducing effective contacts in schools can prevent or delay disease transmissions to other age groups.

### Implementation of Intervention Measures

Next, in order to demonstrate the proposed approach to controlling infectious diseases, we carry out several simulation experiments to examine infectious disease spread under different settings of vaccination and contact reduction. In the case of the 2009 Hong Kong H1N1 influenza epidemic, the government closed all primary schools, kindergartens, and special schools immediately after the first local case was laboratory-confirmed on June 10, 2009 [33]. Besides school closures for contact reduction, the Human Swine Influenza Vaccination Program (HSIVP) were launched on December 21, 2009 [10]. As of early February 2010 (i.e., 50 days after vaccine became available), around 180,000 individuals were administrated vaccine doses, which accounted for around 2.5% of the overall population. For the purpose of demonstration, we explore disease spread under the intervention measures, i.e., *vaccination with a coverage of* 2.5% *of the host population* and *contact reduction within different social settings*. These intervention measures are set to be implemented at the time of day 75.

**Figure 7:**
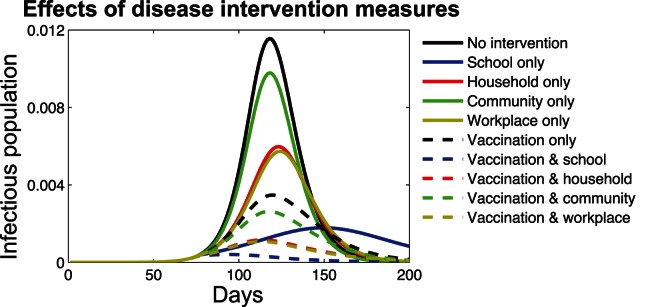
Disease dynamics under the intervention measures of vaccine allocation and contact reduction. Baseline scenario without any intervention (black solid curve); contact reduction only in schools (blue solid curve), households (red solid curve), workplaces (yellow solid curve), and general communities (green solid curve); vaccination only (black dash curve); vaccination and contact reduction in schools (blue dash curve), households (red dash curve), workplaces (yellow dash curve), and general communities (green dash curve).

The simulation results given in Figure 7 have shown that vaccination and contact reduction can meliorate disease prevalence by reducing the incidence rate at the peak of outbreak. In the case of disease intervention by contact reduction only, we can clearly observe from the time course of the infectious population sizes that reducing individuals contacts in different social settings can lead to distinctly different results. Contact reduction in schools (i.e., blue solid curve) outperforms those in the other three social settings, in terms of preventing the occurrence of an infection outbreak and lowering the incidence rate at the peak of disease prevalence. Contact reduction in households (i.e., red solid curve) and workplaces (i.e., yellow solid curve) has a similar effect on disease control and a better performance than that in general communities (i.e., green solid curve). Such a result agrees well with our previous prioritization of social settings for contact reduction, in which schools are identified as the top priority, followed by households and workplaces.

As for the implementation of vaccination and contact reduction simultaneously, the simulation results are shown as the dash curves in Figure 7. With contact reduction in schools, disease spread could be almost eliminated (i.e., blue dash curve). Vaccination when combined with contact reduction in households and workplaces demonstrates an improved performance in reducing disease prevalence than contact reduction in general communities, which is still better than vaccination only. In addition, it is can be observed that the implementation of contact reduction in schools only (i.e., blue solid curve) leads to a lower incidence rate at the peak of disease outbreak than the concurrent implementation of vaccination and contact reduction in general communities (i.e., green solid curve). Meanwhile, the time of disease outbreak is also delayed.

## Discussion

In this work, we have proposed an approach to identifying the relative priorities of subpopulations for some intervention measures, aiming to timely and effectively contain disease transmissions. Specifically, the measures of disease interventions studied here include age-specific vaccine allocation and social setting-specific contact reduction. In an age-structured host population, the contacts among individuals in different subpopulations play a significant role in the dynamics of infectious disease spread. It remains a big challenge to have an accurate and reliable description of individuals contact relationships during the course of disease spread. Previous studies that intended to address the vaccine allocation in an age-structured population mainly relied on the empirical data of individuals cross-age contact frequencies [3], [18], [19], i.e., the contact matrices of individuals reported and physical contacts in several European countries [24]. There are some limitations of such empirical contact matrices: (1) it is not always feasible to have such an empirical description of individuals contact patterns; (2) individuals actual contacts may change during the spread of an infectious disease, which may fail the empirical contact patterns for predicting disease spread. In view of that, Wallinga et al. in [20] proposed a priority identification approach to vaccine allocation that explored the age distribution of new infections in terms of the group-specific force of infection and incidence rates, while individuals contact patterns were not necessary required. However, when considering disease intervention by contact reduction, the problem of individuals contacts still remains unsolved.

In this study, we decomposed individuals actual contacts for disease transmissions into individuals contacts within several specific social settings, i.e., schools, households, workplaces, and general communities. Therefore, the measure of disease intervention by contact reduction can be interpreted as the reduction of the ratio of individuals contacts in a certain social setting. We further inferred the setting-specific contact matrices from the socio-demographical census data of the population. In addition, the combination coefficient of each contact matrix represents the ratio of individuals contacts occurring in that social setting. Thus, the changes of individuals overall contacts can be interpreted as the changes of contact ratios within different social settings. By doing so, we examined the reproduction number for evaluating the effects of implementing intervention measures in containing disease transmissions. Therefore, we can identify the priorities of subpopulations (i.e., age groups and social settings) based on the marginal reduction of the reproduction number corresponding to the changes of susceptible population sizes by age (i.e., vaccination) and contact ratios by social setting (i.e., contact reduction).

We parameterized the proposed approach with the epidemiological data of the 2009 Hong Kong H1N1 influenza epidemic and, thereafter, examined the relative priorities of subpopulations for age-specific vaccination and setting-specific contact reduction in Hong Kong. Our study can be practically applied by public health authorities in preparing and/or assessing their intervention measures for controlling an infectious disease. First, the age distribution of new infections will be always available from the epidemic surveillance system, e.g., the CHP in Hong Kong. Second, the basic patterns of individuals contacts in different social settings will depend mainly on the socio-demographical characteristics of the population, which can be derived either through statistical means or by computational approaches to inferring from census data. Finally, disease control will be more effective when multiple intervention measures are implemented simultaneously.

It should be pointed out that the results from our proposed approach could depend on the accuracy of an age distribution of new infections reported by the surveillance system. Besides, other potential factors that could also affect the results include the report rates of infection that may vary for individuals in different age groups due to their physical and biological conditions, the time needed for the procedure of case confirmation that may lead to a delayed response to disease spread. These factors will be considered in our future studies. In addition, it would be interesting to extend the present study in the following aspects:

- The SIR model provides a basic characterization of disease transmissions. If an extra compartment of latency is taken into account, the model may be enhanced to better fit the real curve of disease spread. Besides, the efficacy of vaccine for inducing immunity and the potential time delay may be incorporated.
- Further to the considered vaccine allocation and contact reduction, other intervention measures that could affect individuals susceptibility (i.e., denoted by 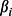) and infectivity (i.e., denoted by 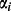), e.g., the prophylactic use of antiviral drugs and the prompt treatment of infections, may also be included. Moreover, the combinations of multiple intervention measures may be analyzed accordingly.
- The costs for different intervention measures may be considered for target identification, which could enable public health authorities to optimize their resource allocation among various options of disease interventions.
